# Nutrigenomics Therapy of Hepatisis C Virus Induced-hepatosteatosis

**DOI:** 10.1186/1471-230X-10-49

**Published:** 2010-05-20

**Authors:** Qing Liu, Stig Bengmark, Shen Qu

**Affiliations:** 1 Department of Endocrinology, Tenth People's Hospital, Tongji University, Shanghai 200072, China; 2 Department of Hepatology, University King's College, London Medical Schools, UK; 3 Beijing YouAn hospital, Beijing 100006, China

## Abstract

Nutrigenomics is a relatively new branch of nutrition science, which aim is to study the impact of the foods we eat on the function of our genes. Hepatosteatosis is strongly associated with hepatitis C virus infection, which is known to increase the risk of the disease progression and reduce the likelihood of responding to anti- virus treatment. It is well documented that hepatitis C virus can directly alter host cell lipid metabolism through nuclear transcription factors. To date, only a limited number of studies have been on the effect of human foods on the nuclear transcription factors of hepatitis C virus -induced hepatosteatosis.

Three nutrients, selected among 46 different nutrients: β-carotene, vitamin D_2_, and linoleic acid were found in a cell culture system to inhibit hepatitis C virus RNA replication. In addition, polyunsaturated fatty acids (PUFAs) especially arachidonic acid (AA), docosahexaenoic acid (DHA), and eicosapentaenoic acid (EPA) have been demonstrated to inhibit hepatitis C virus RNA replication. These PUFAs, in particular the highly unsaturated *n*-3 fatty acids change the gene expression of PPARa and SREBP, suppress the expression of mRNAs encoding key metabolic enzymes and hereby suppress hepatic lipogenesis and triglyceride synthesis, as well as secretion and accumulation in tissues. A recent prospective clinical trial of 1,084 chronic hepatitis C patients compared to 2,326 healthy subjects suggests that chronic hepatitis C patients may benefit from strict dietary instructions.

Increasing evidence suggest that some crucial nuclear transcription factors related to hepatitis C virus -associated hepatosteatosis and hepatitis C virus RNA itself can be controlled by specific anti- hepatitis C virus nutrition. It seems important that these findings are taken into account and specific nutritional supplements developed to be used in combination with interferon as adjunctive therapy with the aim to improve both the early as well as the sustained virological response.

## Introduction

The term nutrigenomics was coined in 1999 by Nancy Fogg-Johnson and her colleague Alex Meroli. The aim of nutrigenomics is to study how various diet ingredients affect the expression of specific genes and hereby provide tools to understand and control the worldwide epidemic of specific chronic diseases, particularly obesity, cancer, cardiovascular disease, diabetes and neurodegenerative diseases such as Alzheimer and Parkinson's disease. These diseases often arise from dysfunctional biological networks, and no single common gene mutation seems responsible, i.e. they appear as polygenic diseases[1-3]. The principle that one gene leads to one protein or one metabolite has in recent years proved to be too simplistic and often incorrect, as demonstrated in experimental studies. Consequently, the traditional "one drug one target" paradigm may not be an effective or the most effective for a successful treatment of polygenic, diet-related diseases. Indeed, the diet, comprised of a multitude of nutritional and chemical molecules, capable of interacting and regulating gene expression and influencing disparate biological processes, has the potential to profoundly influence the Society: disease pattern and health economy. "We are what we eat".

Performing population-scaled epidemiological studies in the absence of genetic knowledge may result in erroneously scientific conclusions and misleading nutritional recommendations. Nutrition research has shifted from epidemiology and physiology to molecular biology and genetics. Nutrition can no longer be viewed as simply epidemiological studies, which aims are to identify relationships between nutrition and chronic disease in genetically uncharacterized populations. Instead nutrigenomics provide tools to look at a deeper level and study the effect of nutrition on molecular and genetic levels and develop/produce diets with the potential to prevent or at least retard the spreading of serious and today widespread chronic diseases including Alzheimer, cancer, and type 2 diabetes. Nutrigenomics offers an exciting area that profoundly will change future direction of not only nutrition, but medicine as a whole, and most likely also Society.

Hepatosteatosis is strongly associated with HCV infection. Recent studies suggest a tight link between HCV infection and hepatic cell lipid metabolism. Several candidate transcription factors with capacity to mediate cross-talks between HCV RNA replication and lipid metabolism have been identified. Since the original observation more than a decade ago that dietary fats act as regulators of gene expression, many transcription factors and prospective targets for fatty acid regulation have been identified. However, so far only a limited number of studies have reported the influence of dietary sources of nutrients on hepatitis C virus (HCV) RNA- induced hepatosteatosis[4]. A recent clinical study comparing 1,084 patients with biopsy-proven HCV-related chronic hepatitis (432 treated with interferon plus ribavirin) with 2,326 healthy subjects, reports a strong association between the composition of diet and severity of HCV-related chronic hepatitis [5] and some dietary components were associated with nonresponse to antiviral therapy. This suggests that HCV patients may benefit from instructions regarding their diet.

The focus of this discussion is the mechanisms of dietary control of gene expression in HCV-induced steatosis, and the aim is to provide practical applications of nutrigenomics, advocating that chronic hepatitis C patients should also receive anti-HCV nutrients as an adjunctive therapy in combination with anti-virus drugs.

## The Link Between HCV Core Protein and Genes Involved in Lipid Metabolism

Although it seems that all genotypes can trigger steatosis, the risk of developing steatosis is significantly higher for people with genotype 3. There is a complex reaction between the genotype 3 virus and liver cells, not seen in other genotypes, giving this group a much higher risk to develop severe steatosis. Around 40% of individuals with hepatitis C have steatosis, compared to between 14% and 31% of the general population. In sharp contrast to this, 60% - 80% of individuals with genotype 3 present with moderate or severe steatosis [6,7].

HCV is an enveloped virus belonging to the Flaviviridae family. The viral genome is a linear, positive-stranded RNA molecule of 9600 nucleotides, that contains a single open reading frame which codes for a polyprotein of 3000 amino acids. The amino-terminal portion of the viral RNA encodes for the structural proteins (C, E1, and E2), followed by the nonstructural proteins (NS2, NS3, NS4A, NS4B, NS5A, and NS5B)[8].

Though the exact mechanism remains elusive, it seems firmly established that hepatitis C virus itself can directly alter host cell cholesterol/lipid metabolism through lipogenetic genes and thereby induce hepatic steatosis. Three transcription genes MTP, PPAR-á and SREBP-1c may involved in HCV interference with lipid metabolism and at three levels: increased triglyceride synthesis, decreased β-oxidation and decreased hepatic export of triglycerides as VLDL.

Microsomal triglyceride transfer protein (MTP) is a major regulator of the assembly and secretion of nascent triglyceride -rich VLDL particles. Perlemuter et al. [9]demonstrate that hepatic overexpression of HCV core protein interferes with the hepatic assembly and secretion of VLDL and inhibits microsomal triglyceride transfer protein (MTP) activity [10]. In support of this observation, some studies have reported a higher prevalence of hypocholesterolemia and hypobetalipoproteinemia in HCV-infected patients compared with control groups [11,12]. Transgenic mice expressing the HCV full-length polyprotein at low levels have decreased plasma triglyceride levels and develop hepatocellular steatosis in the same way as HCV-infected patients[13].

Another well known transcription factor that contributes to de novo fatty acid synthesis in the liver is SREBPs, including three isoforms: SREBP-1a, SREBP-1c and SREBP-2. Although, SREBP-1 and SREBP-2 are structurally similar, their regulation in the liver by nutrients and hormones is quite different. SREBP-1c controls the hepatic and whole body cholesterol and fatty acid synthesis. While SREBP-2 plays a major role in the regulation of cholesterol synthesis and uptake, and the SREBP-1a mainly regulate multiple facets of fatty acid synthesis and VLDL assembly [14,15]. Individual HCV proteins, regardless of HCV genotype, are reported to be able to stimulate lipogenic genes through activation of all three isoforms of SREBPs. Transcription of FAS and SREBP-1c is most likely a main mechanism for HCV-mediated lipogenesis through LXR transcriptional activation. It is yet, however, not fully understood how HCV induces activation of LXR and subsequently SREBP-1c [16].

PPARs are ligand-activated nuclear receptors belonging to the steroid/thyroid hormone receptor superfamily; 3 isoforms designated as α, β/δ, and γ, all of them are involved in lipid homeostasis [17]. PPARα regulates target genes encoding fatty acid-metabolizing enzymes, known to be involved in fatty acid uptake, β-oxidation, transport into peroxisomes, and ω-oxidation of unsaturated fatty acids. Administration of PPARα agonists, such as the widely prescribed fibrate drugs clofibrate, gemfibrozil, and fenofibrate, ameliorate hyperlipidemia in humans and hepatic steatosis in mice [18-20]. However, the association in humans between PPARα function and chronic HCV infection remains a matter of controversy. Rather paradoxical findings are obtained in the transgenic mice. Severe steatosis was unexpectedly observed only in *Ppara*^+/+^:HCVcpTg mice with enhanced fatty acid uptake and decreased mitochondrial β-oxidation due to breakdown of mitochondrial outer membranes. Interestingly, hepatocellular carcinoma developed in approximately 35% of 24-month-old *Ppara*^+/+^:HCVcpTg mice, while tumors were not observed in *Ppara*^+/-^:HCVcpTg, and *Ppara*^-/-^:HCVcpTg mice, suggesting that persistent activation of PPARα is essential for the pathogenesis of hepatic steatosis and HCC induced by HCV infections [21].

HCV proteins associate with insulin resistant (IR). The precise mechanisms whereby HCV induces IR remain elusive, but recent progress has shed light on several critical pathways. Impairment of insulin receptor substrate -1 and insulin receptor substrate -2 expressions have been observed in the livers of HCV infected patients as well as in HCV core transgenic mice. Specifically, HCV core protein has been shown to inhibit insulin induced phosphorylation of the p85 subunit of phosphatidylinositol 3-kinase (PI3K) and Akt, which are downstream components of insulin receptor substrate in the liver [22].

## Dietary FFA Composition Inhibit HCV Replication and Hcv Induced- Lipogenesic Genes

Hepatic free fatty acids (FFAs) are de novo synthesized within the hepatocytes, released by adipose tissue and taken up by the liver, or generated in the liver by the hydrolysis of chylomicrons from the intestine. The findings indicate that changes in the fatty acid composition of chylomicron remnants can alter the rate of their uptake by the liver. It seems increasingly accepted that the fat composition of diet not only constitute substrate for energy metabolism, and are important for membrane formation and expression of signaling molecules, but also regulates gene expression[23], including hepatic fatty acid-regulated transcription factors (PPARα, SREBP-1, ChREBP and MLX). Since the steatosis and insulin resistance are closely linked to the progression of liver disease in HCV infected patients [24], such regulatory schemes will impact whole body metabolism and contribute to onset and progression of HCV infection, and more importantly the facilitation of early virological response (EVR) or a sustained viro-logical response (SVR).

### Dietary fatty acid inhibit HCV RNA replication

This is partly due to the lack of suitable animal model, a cell culture system (OR6 assay system) has been used to study the efficient of HCV RNA replication, and shown to be hampered by anti-HCV nutrients[25-28]. The study examined comprehensively 46 different nutrients from four nutrient groups: vitamins, amino acids, fatty acids, and salts. Three nutrients--β-carotene, vitamin D_2_, and linoleic acid were found to inhibit HCV RNA replication and combination of the three caused additive and/or synergistic effects on HCV RNA replication. Furthermore, combined treatment with each of the three nutrients and interferon alpha or beta or fluvastatin inhibited in an additive manner HCV RNA replication. In contrast, Vitamin E was found to enhance HCV RNA replication and negated the effects of the three anti-HCV nutrients and cyclosporine, in contrast to those of interferon or fluvastatin[29-32].

Also PUFAs have demonstrated the ability to inhibit HCV RNA replication. It was observed in one study that several PUFAs including arachidonic acid (AA), docosahexaenoic acid (DHA), and eicosapentaenoic acid (EPA) had the capacity to exert anti-HCV activities using an HCV subgenomic RNA replicon system. PUFAs, such as, arachidonic acid, EPA, and DHA, inhibited HCV RNA replication already on the first day after initiation: arachidonic acid (≈4.5-fold (*P *= 0.0005), EPA (≈3-fold (*P *= 0.0047), and DHA (≈6.4-fold (*P *= 0.0002). In contrast, saturated (lauric, myristic, and palmitic) and also monounsaturated (oleic) fatty acids induced HCV RNA replication, observed as early as 4 days after initiation of treatment. When AA was combined with IFN-α, strong synergistic anti-HCV effect was observed as demonstrated by an isobologram analysis [33,34]. Interestingly, this study suggested that the PUFAs inhibit HCV RNA replication by a mechanism independent of their ability to inhibit lipogenic gene expression - by antagonizing LXR-SREBP-1c Pathway.

The precise mechanism underlying the anti-HCV activities of the nutrients are, however, not fully understood and further studies needed to clarify the targets of the nutrients responsible for their anti-HCV activities.

### Dietary FFA composition affects HCV induced- Lipogenesic genes

Studies with various dietary fatty acids have revealed several major metabolic pathways that are targeted by PUFA, each pathway involving changes in gene expression [35-37]. PPAR and SREBP-1c genes, known to induce fatty acid oxidation and synthesis respectively are key targets for PUFA control of hepatic gene expression. *n-*3 PUFAs have rapid effects on gene expression, the changes in mRNAs encoding several lipogenic enzymes can be detected within hours of feeding animals diets enriched in *n-*3 PUFA. Moreover, these effects are sustained as long as the *n-*3 PUFAs remain in the diet. Two general mechanisms characterize fatty acid as a regulator of gene expression:

1. Fatty acids bind directly to the transcription factor and control transcription factor activities, such as PPAR(α, β, γ1 & γ2), HNF-4 (α & γ), RXRα and LXRα. In this fashion, fatty acids act like hydrophobic hormones regulating the function of nuclear receptors and their impact on transcriptional processes.

2. Fatty acids control the nuclear abundance of key transcription factors, such as SREBP-1, NFκB, ChREBP and MLX[38].

PPAR subtypes are the most widely accepted fatty acid-regulated transcription factors. Certain fatty acids, however, are better than others at activating PPAR. Twenty-carbon PUFA is an important determinant in the control of PPAR activity and its target genes. PPARα binds 20:5*n*-3, but not 18:1*n*9, and activates PPAR in rat primary hepatocytes [39]. Activated PPAR induced- lipoprotein lipase and fatty acid transporters (CD36) and enhance adipocyte differentiation, inhibition of NFB function and cytokine and expression of COX-2 [40,41]. The glitazones, *e.g*. troglitazone, pioglitazone, and rosiglitazone, are pharmacological PPAR agonists and are used in the treatment of insulin resistance. Pharmacological activation of PPAR and PPAR reduces lipid levels in muscle and adipose tissue and improves insulin sensitivity in these tissues [42,43]. Although *n-*3 PUFAs are weak agonists of PPARs compared with pharmacological agonists, it has a significant effect on insulin sensitivity in various tissues, particularly skeletal muscle [44]. Thus, *n*-3 PUFA action on insulin responsiveness in these tissues may extend beyond its regulation of PPAR activity.

n-3 and n-6 PUFA are well-established suppressors of mRNA SREBP-1 abundance, but not SREBP-2, through inhibit SREBP-1 gene transcription, induce mRNA_SREBP-1 _instability and inhibit SREBP processing. It has been demostrated that 22:6, n-3, but not 20:4, n-6, is a major regulator of nuclear SREBP-1 abundance and target genes[45]. The ability of polyunsaturated fatty acids to inhibit SREBP conversion from its inactive to its active form relates to both physical and biochemical effects[46]. The physical mechanism is that addition of fatty acids to these model membranes decreases the affinity of cholesterol for phospholipid and this in turn results in enhanced transfer from cholesterol-rich regions (such as the plasma membrane) to cholesterol-poor regions (such as the endoplasmic reticulum, leading to decreased SREBP transport out of the endoplasmic reticulum to the Golgi apparatus [47]. Another possible mechanism by which polyunsaturated fatty acids decrease SREBP is by changing the cellular composition of membranes. A rather recent study showed that polyunsaturated fatty acids increase the hydrolysis of plasma membrane sphingomyelin to ceramide[48]. Lower amounts of sphingomyelin result in decreased ability to solubilize free cholesterol, which leads to intracellular displacement of cholesterol and a consequent decrease in SREBP-mediated gene transcription[49]. Ceramide itself is through effects on sphingolipid synthesis as a potent inhibitor of SREBP processing [50], most likely contributing to regulation of endoplasmic reticulum-Golgi vesicular transport [51]. All this information supports the role and interaction of polyunsaturated fatty acids in the different steps of sphingolipid metabolism that affect SREBP processing.

In vivo and vitro studies demonstrate that PUFAs have the capacity to control hepatic gene expression and hepatic lipid composition and hereby affect whole-body lipid composition through regulating PPAR- and SREBP-1c genes. However, more recent studies using a MCD diet-fed model of steatohepatitis suggest that hepatic lipid peroxidation correlate positively with the amount of dietary PUFAs.

Because unsaturated fatty acids are excellent substrates for lipid peroxidation, and because hepatic lipid peroxidation is a prominent feature of MCD-related liver disease, unsaturated fat in the MCD formula are usually regarded to play an important role in *MCD*-mediated hepatotoxicity. The standard MCD formula contains 10% corn oil, which is highly enriched in unsaturated fat. To test the theory, MCD formulas with varying amounts of unsaturated fat content from 2% to 59% were prepared and their effects on steatosis, lipid peroxidation, and liver injury were studied [52-55]. The results indicated that hepatic lipid peroxidation is directly related to the amount of unsaturated fat in the MCD diet, and that lipid peroxidation correlates with hepatic induction of proinflammatory cytokine genes and hepatic inflammation.

Corn oil, the one of the most noxious fats was incorporated into the MCD formula, is rich in n-6 fatty acids. High intake of n-6 fatty acids is known to be implicated in the pathogenesis of nonalcoholic steatohepatitis in humans. In contrast, n-3 fatty acids are reported to improve nonalcoholic fatty liver disease.

A recent study addressed the role of n-3 fatty acids in the MCD model of steatohepatitis[56,57]. Feeding an n-3 PUFA-enriched diet failed to prevent lipotoxic hepatocellular injury and inflammatory recruitment, although activated PPAR alpha and suppressed hepatic de novo lipogenesis. The researchers concluded: "Instead, the very high levels of hepatic lipoperoxides may have abrogated the protection that would otherwise be conferred by PPAR alpha activation, and could also be responsible for lipotoxic hepatocellular injury and inflammatory recruitment."

## Conclusions

Approximately 170 million people are infected worldwide with HCV, making the condtion a major global health problem. The combination of pegylated interferon (IFN) with ribavirin is currently the most effective therapy for chronic HCV hepatitis, and long-term treatment has been shown to improve the sustained virological response (SVR) rate. However, the SVR rate still remains at approximately 55% and patients with HCV genotype 1 infection combined with steatosis are significantly less likely to achieve a week-12 early virological response (EVR) or a sustained viro-logical response (SVR).

Nutrigenomics offer hope in the search for novel therapeutic and nutritional management options. Dietary FFA composition can inhibit both HCV replication and HCV induced- lipogenesic genes. Three nutrients, β-carotene, vitamin D2, and linoleic acid are found to inhibit HCV RNA replication and their combination to cause additive and/or synergistic effects on HCV RNA replication. PUFAs including AA, DHA and EPA are also demonstrated to inhibit HCV RNA replication, while saturated (lauric, myristic, and palmitic) and monounsaturated (oleic) fatty acids induce HCV RNA replication.

PPAR and SREBP-1c gene, known to induce fatty acid oxidation and synthesis respectively, are key targets for PUFA control of hepatic gene expression. *n*-3 PUFAs are weak agonists of PPARs compared with pharmacological agonists, but has a significant effect on insulin sensitivity in various tissues, particularly skeletal muscle. These results provide useful information for improvement of the SVR rates of patients receiving the currently standard IFN therapy. In addition, these findings may contribute to the development of nutritional supplements of use in the treatment of people with chronic hepatitis C.

## Abbreviations

HCV: Hepatitis C viruse; FFA: Free fatty acids; MTP: Microsomal triglyceride transfe protein; PPARs: Peroxisome Proliferator-Activated Receptors; SREBs: Sterol Regulatory Element Binding Proteins; PUFAs: Polyunsaturated fatty acids; MCD: methionine and choline-deficient diet.

## Competing interests

The authors declare that they have no competing interests.

## Authors' contributions

Liu Qing, Stig Bengmark and Qu Shen participated in the design of the review. Liu qing and Qu Shen draft and Stig Bengmark revise the manuscript. All authors read and approved the final manuscript.

## Pre-publication history

The pre-publication history for this paper can be accessed here:

http://www.biomedcentral.com/1471-230X/10/49/prepub
